# Construction Notes

**Published:** 1894-04-21

**Authors:** 


### CONSTRUCTION NOTES.

### Re-opening Nurses' Institute, Bedford.

The ceremony of re-opening this establishment was performed recently, the Duchess of Bedford officiating. The institute is carried on in connection with the dispensary, which building has been undergoing enlargement in order to accommodate the increased nursing staff that was required. The additional height to the dispensary has added to the imposing appearance of the elevation of this building. The new story has been added without removing the roof, which was carefully raised, stayed, and braced. The internal arrangements have been remodelled to meet the present requirements. The entrance is approached under a covered glass way, the floor being laid with encaustic tiles. Convenient to the entrance is the nurses' luggage-room, and beyond this the kitchen and offices. The kitchen range has been fitted up with Wilson's patent fire and boiler by Wilson's Economic Fire Company. On the first floor are the matron's and nurses' sitting-rooms, and also the matron's bed-room, two nurses' bed-rooms, bath-room, and offices; the dining-room, which is also on the first floor, is fitted with a lift down to the kitchen. On the second floor are six nurses' bed-rooms. In the dispensary department the surgery has been enlarged in order to obtain the necessary room and increase of shelf space. The work has been carried out by the contractor, Mr. Samuel Foster, of Kempston, from the plans prepared by Messrs. Usher and Anthony, architects and surveyors, of St. Paul's Square, and under their immediate personal superintendence.

### Maternity Hospital Aberdeen Dispensary.

The new maternity hospital provided in connection with the Aberdeen Dispensary was recently opened. The new hospital, which is closely adjacent to the dispensary buildings, is a most cheery and comfortably-appointed little building, with more of the aspect of a home than an hospital, and with the brightest prospect of usefulness for the community. Sir William Henderson, in the absence of the Lord Provost, performed the opening ceremony. He explained that the establishment would be carried out under the superintendence of the Professor of Midwifery at the University of Aberdeen, and with the services of Miss Jasper as matron. The hospital is planned on the ground floor to contain the committee-room. On the first floor a ward with four beds, but large enough for six beds, matron's store-room. Second floor, bed-rooms, bath-room, small ward for isolating and other purposes, matron's sitting-room, &c. There is a large washhouse well fitted up. The whole establishment, which has been fitted up most comfortably, has cost the managers about £250 up to date, the maintenance being reckoned to involve an annual expenditure of about £200. Mr. Henderson, of Union Street, Aberdeen, is both architect and contractor of this new building.

